# Antimicrobial Resistance and ESBL-Associated Predictors Among Uropathogens: A 2019–2024 Isolate-Level Study

**DOI:** 10.3390/antibiotics15030323

**Published:** 2026-03-23

**Authors:** Raul-Lucian Ene, Roxana Popescu, Aurica Elisabeta Cobec, Daniela Puscasiu, Ileana-Adriana Ene, Daliborca Cristina Vlad, Ionut Marcel Cobec, Peter Seropian

**Affiliations:** 1 Doctoral School, Faculty of Medicine, “Victor Babes” University of Medicine and Pharmacy Timisoara, 300041 Timisoara, Romania; 2 Department of Urology, Emergency Hospital Petrosani, 332019 Petrosani, Romania; 3 ANAPATMOL Research Center, Faculty of Medicine, “Victor Babes” University of Medicine and Pharmacy Timisoara, 300041 Timisoara, Romania; 4 Department of Cell and Molecular Biology, “Victor Babes” University of Medicine and Pharmacy Timisoara, 300041 Timisoara, Romania; 5 Clinic of Internal Medicine-Cardiology, Klinikum Freudenstadt, 72250 Freudenstadt, Germany; 6 Diaverum Petroșani Nephrology and Dialysis Medical Center, 332019 Petrosani, Romania; 7 Department of Pharmacology, Faculty of Medicine, “Victor Babes” University of Medicine and Pharmacy Timisoara, 300041 Timisoara, Romania; 8 Department of Obstetrics and Gynecology, Faculty of Medicine, Medical Center-University of Freiburg, 79106 Freiburg, Germany; 9 Clinic of Obstetrics and Gynecology, Klinikum Freudenstadt, 72250 Freudenstadt, Germany

**Keywords:** urinary tract infections, antimicrobial resistance, uropathogens, ESBL, antimicrobial susceptibility, isolate-level analysis

## Abstract

**Background/Objectives:** Urinary tract infections (UTIs) are among the most common bacterial infections and represent a major source of antimicrobial use. Increasing antimicrobial resistance among uropathogens, particularly the emergence of extended-spectrum beta-lactamase (ESBL)-producing organisms, complicates empiric treatment strategies. ESBL-producing organisms are clinically relevant because they are frequently associated with multidrug resistance and significantly limit empiric antimicrobial treatment options in urinary tract infections. The study period starting in 2019 was selected to reflect contemporary resistance patterns and to ensure consistency with the updated EUCAST antimicrobial susceptibility interpretation criteria introduced at that time. This study aimed to characterize antimicrobial resistance patterns among uropathogens isolated from lower UTIs and to identify independent predictors of antimicrobial resistance using isolate-level analyses. **Methods:** This retrospective observational study included 1470 patients and isolates with clinically suspected lower UTIs who underwent urine culture and antimicrobial susceptibility testing between 2019 and 2024 at a single clinical center. Antimicrobial susceptibility was interpreted according to European Committee on Antimicrobial Susceptibility Testing (EUCAST) criteria, and ESBL production was assessed among Gram-negative (GN) isolates. Multivariable generalized estimating equation (GEE) logistic regression models accounting for patient clustering were used to identify predictors of resistance. **Results:** A total of 1470 patients and isolates were included. *Escherichia coli* was the most frequent uropathogen (66.0%), followed by *Klebsiella pneumoniae* and *Enterococcus faecalis*. Among Gram-negative isolates, 17.3% were ESBL-positive. Resistance rates were highest for ciprofloxacin (35.4%) and trimethoprim/sulfamethoxazole (31.7%), while fosfomycin and nitrofurantoin retained high activity against *E. coli*. In multivariable analyses, ESBL production was the strongest independent predictor of resistance to several antimicrobials, including ciprofloxacin (aOR 9.83), amoxicillin/clavulanic acid (aOR 3.22), trimethoprim/sulfamethoxazole (aOR 2.89), and cefotaxime (aOR 1337). Pathogen identity was also independently associated with resistance. **Conclusions:** Antimicrobial resistance among uropathogens was heterogeneous and predominantly driven by pathogen identity and ESBL production. ESBL status emerged as the most consistent and powerful predictor of resistance across multiple antimicrobials, underscoring its clinical relevance for empiric treatment decisions and antimicrobial stewardship in urinary tract infections.

## 1. Introduction

Urinary tract infections (UTIs) represent one of the most common bacterial infections encountered in clinical practice and account for a substantial proportion of antimicrobial prescriptions worldwide. They affect individuals across all age groups, with higher incidence among women, older adults, and patients with comorbidities. Although many UTIs are community-acquired and clinically uncomplicated, their management has become increasingly challenging due to the global rise in antimicrobial resistance among uropathogens [1,2,3].

The epidemiology of uropathogens has remained relatively stable over time, with *E. coli* consistently identified as the predominant causative agent, followed by *K. pneumoniae*, *E. faecalis*, *Proteus mirabilis*, and other Gram-negative and Gram-positive organisms. However, while pathogen distribution has changed only modestly, antimicrobial susceptibility profiles have evolved considerably, with increasing antimicrobial resistance reported to commonly used oral agents such as fluoroquinolones, trimethoprim/sulfamethoxazole, and beta-lactam antimicrobials. These trends have significant implications for empiric therapy, particularly in outpatient and ambulatory care settings where microbiological data are often unavailable at treatment initiation [4,5,6].

Among antimicrobial resistance mechanisms, the emergence and spread of extended-spectrum beta-lactamase (ESBL)-producing Gram-negative bacteria represents a major public health concern. ESBL enzymes confer antimicrobial resistance to third- and fourth-generation cephalosporins as well as monobactams, and are frequently associated with multidrug-resistant phenotypes, limiting therapeutic options and increasing the likelihood of treatment failure. Although ESBL production has traditionally been associated with healthcare-associated infections, recent years have seen a growing prevalence of ESBL-producing uropathogens in community settings, blurring the distinction between community- and healthcare-associated antimicrobial resistance patterns. Identifying predictors associated with ESBL-producing uropathogens is clinically important because these organisms are frequently resistant to multiple antibiotic classes, which significantly limits empiric treatment options and may increase the risk of therapeutic failure [7,8,9].

In Romania and other Central and Eastern European countries, several studies have reported increasing antimicrobial resistance among uropathogens, including rising prevalence of ESBL-producing Enterobacterales in both hospital and community settings. These regional trends highlight the importance of local epidemiological data to guide empiric antimicrobial therapy and antimicrobial stewardship strategies [10,11].

Previous studies have described antimicrobial resistance rates among uropathogens and have reported associations between antimicrobial resistance and factors such as age, sex, prior antimicrobial exposure, healthcare contact, and pathogen species. However, many analyses rely on aggregated susceptibility data or patient-level summaries and do not adequately account for the complex structure of microbiological datasets, where multiple isolates and multiple antimicrobial tests may be linked to the same patient. Furthermore, the independent contribution of ESBL production to antimicrobial resistance against non–beta-lactam antimicrobials, such as fluoroquinolones or trimethoprim/sulfamethoxazole, remains incompletely characterized in routine clinical populations [10,11,12,13].

In this context, isolate-level analyses that incorporate detailed microbiological data and appropriate statistical methods are essential to disentangle pathogen-specific effects from patient-related factors and antimicrobial resistance mechanisms. Multivariable approaches that account for within-patient correlation may provide more accurate estimates of predictors of antimicrobial resistance and improve understanding of how ESBL status interacts with demographic and microbiological variables [14,15,16].

The present study aimed to characterize antimicrobial susceptibility patterns among uropathogens isolated from patients with lower urinary tract infections over a six-year period and to evaluate the prevalence and distribution of ESBL production among Gram-negative isolates. In addition, we sought to identify independent predictors of antimicrobial resistance to selected clinically relevant antimicrobials using isolate-level analyses and multivariable models accounting for patient clustering. By integrating susceptibility profiles, ESBL status, and minimum inhibitory concentration data, this study provides a comprehensive isolate-level assessment of antimicrobial resistance determinants in a real-world clinical setting [17,18,19].

## 2. Results

### 2.1. Descriptive Analysis of Patient Characteristics and Uropathogen Distribution

A total of 1470 patients diagnosed with urinary tract infections were included from Petroșani Emergency Hospital during the study period. Patient-level analysis was performed, with one record corresponding to each individual (Table 1). The median age of the study population was 66 years (Interquartile Range—IQR: 54–75). Age distribution deviated significantly from normality (Shapiro–Wilk test: W = 0.914, *p* < 0.001); therefore, age was summarized using median and interquartile range. Of the 1470 patients, 964 (65.6%) were female and 506 (34.4%) were male. Thus, female patients accounted for approximately two-thirds of the cohort. Regarding the environment of origin, 1335 patients (90.8%) were from urban areas, whereas 135 patients (9.2%) were from rural areas.

**Figure 1:**
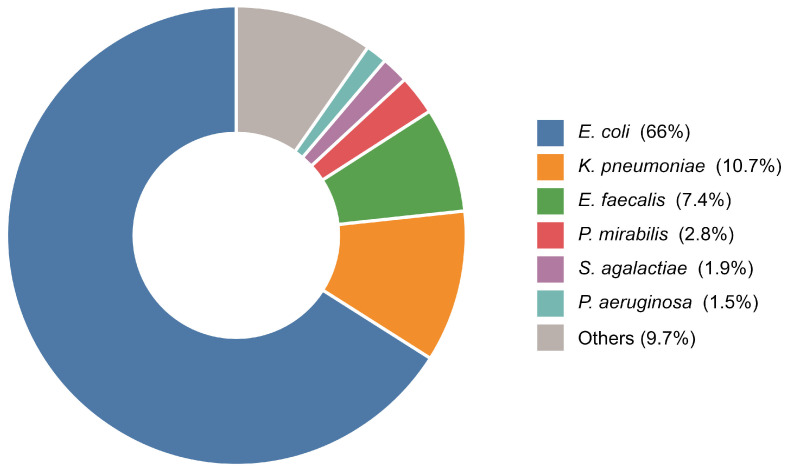
Frequency distribution of uropathogens isolated from patients with urinary tract infections.

Of the 1470 isolates, *E. coli* was identified in 970 cases (66.0%), representing the most frequent uropathogen. *K. pneumoniae* was isolated in 157 cases (10.7%), while *E. faecalis* accounted for 109 isolates (7.4%). Less frequent pathogens included *P. mirabilis* with 41 isolates (2.8%), *Streptococcus agalactiae* with 28 isolates (1.9%), and *Pseudomonas aeruginosa* with 22 isolates (1.5%). Each of these organisms accounted for less than 3% of the total isolates. All remaining pathogens were identified in fewer than 20 isolates each and are reported in Supplementary Table S1. As shown in Figure 1, the isolate distribution was highly concentrated, with a single pathogen accounting for approximately two-thirds of all isolates, followed by a progressive decrease in frequency across remaining species.

### 2.2. Antimicrobial Susceptibility Patterns of Major Uropathogens

Table 2 presents the antimicrobial susceptibility profile of selected clinically relevant antimicrobials, including first-line agents, commonly used empiric therapies, and antimicrobials with high resistance or high susceptibility rates. Antimicrobials with limited clinical relevance or low testing frequency are not shown in the main table; the complete susceptibility dataset is provided in Supplementary Table S2.

Among fluoroquinolones, ciprofloxacin showed susceptibility in 899/1443 tests (62.3%) and antimicrobial resistance in 511/1443 tests (35.4%). High antimicrobial resistance was observed for norfloxacin, with 548/615 tests (89.1%) classified as resistant, while levofloxacin demonstrated antimicrobial resistance in 94/223 tests (42.2%). Among commonly used oral agents, amoxicillin/clavulanic acid was susceptible in 894/1280 tests (69.8%), with antimicrobial resistance observed in 233/1280 tests (18.2%). Trimethoprim/sulfamethoxazole showed antimicrobial resistance in 404/1275 tests (31.7%), whereas nitrofurantoin and fosfomycin retained higher activity, with susceptibility rates of 77.5% (955/1233) and 97.7% (1170/1197), respectively. Among beta-lactam antimicrobials frequently used in clinical practice, susceptibility rates were 76.4% (981/1284) for cefotaxime, 83.5% (469/562) for ceftriaxone, 85.1% (1087/1278) for cefepime, and 81.7% (1058/1295) for ceftazidime. Aminoglycosides demonstrated high susceptibility, with gentamicin susceptible in 1077/1317 tests (81.8%) and amikacin in 1189/1291 tests (92.1%). Carbapenems showed the highest susceptibility rates overall, with ertapenem susceptible in 1226/1242 tests (98.7%), meropenem in 1245/1292 tests (96.4%), and imipenem in 1181/1276 tests (92.6%).

Among *E. coli* isolates, fosfomycin demonstrated susceptibility in 1068/1072 tests (99.6%), while nitrofurantoin was susceptible in 879/946 tests (92.9%), with antimicrobial resistance observed in 1.2% (11/946) of tests.

Among commonly used oral agents, amoxicillin/clavulanic acid showed susceptibility in 763/997 tests (76.5%) and antimicrobial resistance in 105/997 tests (10.5%). Trimethoprim/sulfamethoxazole demonstrated antimicrobial resistance in 278/949 tests (29.3%), while ciprofloxacin antimicrobial resistance was observed in 272/973 tests (28.0%).

Third- and fourth-generation cephalosporins retained substantial activity against *E. coli*. Susceptibility rates were 84.8% (824/972) for cefotaxime, 91.7% (387/422) for ceftriaxone, 92.9% (895/963) for cefepime, and 90.3% (879/973) for ceftazidime. Cefuroxime demonstrated susceptibility in 588/724 tests (81.2%), with antimicrobial resistance in 18.1% (131/724).

Aminoglycosides showed high activity, with gentamicin susceptible in 865/973 tests (88.9%) and amikacin in 934/973 tests (96.0%). Carbapenems demonstrated near-universal susceptibility. Meropenem and imipenem were susceptible in 99.9% of tests (970/971 and 969/970, respectively), while ertapenem showed susceptibility in 969/970 tests (99.9%).

The full antimicrobial susceptibility profile for *E. coli*, including less frequently tested and reserve antimicrobials, is provided in Supplementary Table S3.

Among *K. pneumoniae* isolates, susceptibility to commonly used antimicrobials was markedly lower compared with *E. coli*. Among oral agents, amoxicillin/clavulanic acid showed susceptibility in 93/162 tests (57.4%), with antimicrobial resistance in 51/162 tests (31.5%). Trimethoprim/sulfamethoxazole demonstrated antimicrobial resistance in 68/160 tests (42.5%), while ciprofloxacin antimicrobial resistance was observed in 73/160 tests (45.6%). Third- and fourth-generation cephalosporins showed reduced activity. Susceptibility rates were 48.8% (78/160) for cefotaxime, 51.2% (82/160) for ceftazidime, 55.0% (88/160) for cefepime, and 60.0% (39/65) for ceftriaxone. Cefuroxime demonstrated susceptibility in 27/91 tests (29.7%), with antimicrobial resistance in 69.2% (63/91).

Aminoglycosides retained higher activity, with gentamicin susceptible in 102/160 tests (63.8%) and amikacin in 135/160 tests (84.4%). Carbapenems remained the most active agents. Imipenem and meropenem showed susceptibility in 83.8% (134/160) and 85.0% (136/160) of tests, respectively, while ertapenem demonstrated susceptibility in 92.3% (132/143), with antimicrobial resistance observed in 5.6% (8/143). Piperacillin/tazobactam showed susceptibility in 98/157 tests (62.4%), with antimicrobial resistance in 44/157 tests (28.0%). Fosfomycin retained activity, with susceptibility in 63/75 tests (84.0%).

The complete antimicrobial susceptibility profile for *K. pneumoniae*, including less frequently tested and reserve antimicrobials, is provided in Supplementary Table S4.

All *E. faecalis* isolates demonstrated full susceptibility to ampicillin, with 109/109 tests (100.0%) classified as susceptible. Similarly, vancomycin and teicoplanin showed susceptibility in 107/107 (100.0%) and 109/109 (100.0%) tests, respectively. High susceptibility was also observed for tigecycline, with 108/108 tests (100.0%) classified as susceptible. Linezolid retained activity, with susceptibility in 104/109 tests (95.4%), while antimicrobial resistance was observed in 2/109 tests (1.8%).

High-level aminoglycoside antimicrobial resistance was frequently observed. High-level gentamicin antimicrobial resistance was present in 61/108 tests (56.5%), while high-level streptomycin antimicrobial resistance was identified in 56/108 tests (51.9%), indicating reduced potential for synergistic therapy in more than half of isolates. Among fluoroquinolones, ciprofloxacin susceptibility was observed in 44/103 tests (42.7%), with antimicrobial resistance identified in 58/103 tests (56.3%).

The complete antimicrobial susceptibility profile for *E. faecalis*, including uniformly resistant and infrequently tested antimicrobials, is provided in Supplementary Table S5.

### 2.3. Extended-Spectrum Beta-Lactamase (ESBL) Production Among Gram-Negative Uropathogens

**Figure 2:**
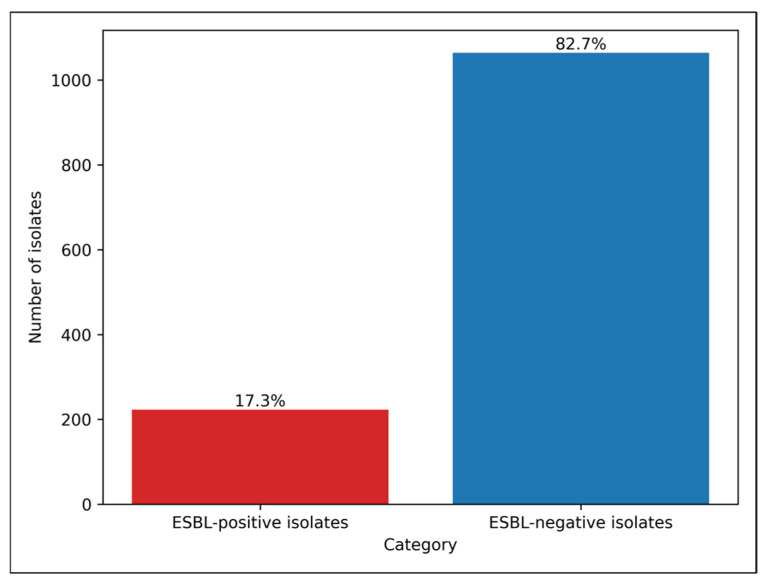
Overall prevalence of extended-spectrum beta-lactamase (ESBL) production among Gram-negative uropathogens.

Among 1287 Gram-negative isolates, 223 isolates (17.3%) were identified as ESBL-positive, while 1064 isolates (82.7%) were ESBL-negative. Thus, approximately one in six Gram-negative uropathogens demonstrated ESBL production in the study population. The results are summarized in Figure 2.

Only Gram-negative pathogens are reported in Supplementary Table S6.

ESBL prevalence differed significantly between pathogens (Chi-square test with Monte Carlo simulation, *p* < 0.001).

Among *E. coli*, 165 of 970 isolates (17.0%) were ESBL-positive. A higher proportion was observed among *K. pneumoniae*, with 57 of 157 isolates (36.3%) classified as ESBL-positive.

No ESBL-producing isolates were identified among *P. mirabilis* (0/41) or *P. aeruginosa* (0/22).

Among Gram-negative isolates originating from urban environments, 197 of 1162 isolates (17.0%) were ESBL-positive. In rural environments, 26 of 125 isolates (20.8%) were ESBL-positive.

The difference in ESBL prevalence between urban and rural isolates was not statistically significant (Chi-square test, *p* = 0.280), indicating comparable ESBL distribution across environments in this cohort. The results are presented in Table 3.

### 2.4. Univariate Analysis of Factors Associated with Antimicrobial Resistance

Univariate analyses were performed to identify factors associated with antimicrobial resistance to selected antimicrobials at the isolate level. The antimicrobials included in this analysis—ciprofloxacin, trimethoprim/sulfamethoxazole, amoxicillin/clavulanic acid, and cefotaxime—were chosen based on their frequent use in the treatment of urinary tract infections and their clinical relevance for empiric and targeted therapy. These analyses aimed to explore the associations between antimicrobial resistance and selected demographic, microbiological, and antimicrobial resistance-related factors prior to multivariable modeling.

In univariate analysis, several factors were significantly associated with ciprofloxacin antimicrobial resistance at the isolate level (Table 4). Antimicrobial resistance was more frequent among isolates from male patients, with an odds ratio (OR) of 1.77 (95% Confidence Interval-CI: 1.404–2.231, *p* < 0.001) compared with isolates from female patients.

No significant association was observed between ciprofloxacin antimicrobial resistance and environment of origin. Isolates from rural settings had an OR of 1.15 (95% CI: 0.781–1.688, *p* = 0.449) compared with urban isolates.

Among Gram-negative isolates, ESBL production was strongly associated with ciprofloxacin antimicrobial resistance. ESBL-positive isolates had an OR of 8.33 (95% CI: 5.954–11.778, *p* < 0.001) compared with ESBL-negative isolates.

Pathogen-specific analyses (restricted to pathogens with N ≥ 20) demonstrated significantly higher odds of ciprofloxacin antimicrobial resistance compared with *E. coli*. Antimicrobial resistance was more frequent in *E. faecalis* (OR 3.31, 95% CI: 2.187–5.003, *p* < 0.001), *K. pneumoniae* (OR 2.12, 95% CI: 1.502–2.989, *p* < 0.001), *P. mirabilis* (OR 4.95, 95% CI: 2.557–9.581, *p* < 0.001), and *P. aeruginosa* (OR 11.5, 95% CI: 3.873–34.431, *p* < 0.001).

In univariate analysis, several factors were associated with antimicrobial resistance to trimethoprim/sulfamethoxazole at the isolate level (Table 5). Antimicrobial resistance was more frequent among isolates from male patients, with an odds ratio (OR) of 1.293 (95% CI: 0.999–1.672, *p* = 0.043) compared with isolates from female patients.

No association was observed between trimethoprim/sulfamethoxazole antimicrobial resistance and environment of origin. Isolates from rural settings had an OR of 0.996 (95% CI: 0.650–1.506, *p* = 0.985) compared with urban isolates.

Among Gram-negative isolates, ESBL production was significantly associated with trimethoprim/sulfamethoxazole antimicrobial resistance. ESBL-positive isolates had an OR of 2.882 (95% CI: 2.116–3.929, *p* < 0.001) compared with ESBL-negative isolates.

Pathogen-specific analyses (restricted to pathogens with N ≥ 20) demonstrated increased odds of antimicrobial resistance compared with *E. coli*. Antimicrobial resistance was higher in *K. pneumoniae* (OR 1.789, 95% CI: 1.266–2.528, *p* < 0.001) and particularly pronounced in *P. mirabilis* (OR 9.912, 95% CI: 4.521–21.730, *p* < 0.001).

In univariate analysis, several factors were significantly associated with antimicrobial resistance to amoxicillin/clavulanic acid at the isolate level (Table 6).

Antimicrobial resistance was more frequent among isolates from male patients, with an odds ratio (OR) of 2.359 (95% CI: 1.739–3.199, *p* < 0.001) compared with isolates from female patients.

No significant association was observed between antimicrobial resistance and environment of origin. Isolates from rural settings had an OR of 1.163 (95% CI: 0.692–1.892, *p* = 0.531) compared with urban isolates.

Among Gram-negative isolates, ESBL production was significantly associated with amoxicillin/clavulanic acid antimicrobial resistance. ESBL-positive isolates had an OR of 2.230 (95% CI: 1.571–3.146, *p* < 0.001) compared with ESBL-negative isolates.

Pathogen-specific analyses (restricted to pathogens with N ≥ 20) demonstrated substantially increased odds of antimicrobial resistance compared with *E. coli*. Antimicrobial resistance was higher in *K. pneumoniae* (OR 3.929, 95% CI: 2.652–5.820, *p* < 0.001) and *P. mirabilis* (OR 4.404, 95% CI: 2.065–9.394, *p* < 0.001).

In univariate analysis, several factors were significantly associated with antimicrobial resistance to cefotaxime at the isolate level (Table 7).

Antimicrobial resistance was more frequent among isolates from male patients, with an odds ratio (OR) of 2.215 (95% CI: 1.679–2.923, *p* < 0.001) compared with isolates from female patients.

No significant association was observed between cefotaxime antimicrobial resistance and environment of origin. Isolates from rural settings had an OR of 1.275 (95% CI: 0.808–1.975, *p* = 0.259) compared with urban isolates.

Among Gram-negative isolates, ESBL production showed a very strong association with cefotaxime antimicrobial resistance. ESBL-positive isolates had an OR of 91.142 (95% CI: 55.338–156.710, *p* < 0.001) compared with ESBL-negative isolates.

Pathogen-specific analyses (restricted to pathogens with N ≥ 20) demonstrated markedly increased odds of antimicrobial resistance compared with *E. coli*. Antimicrobial resistance was higher in *K. pneumoniae* (OR 5.810, 95% CI: 4.058–8.317, *p* < 0.001) and *P. mirabilis* (OR 4.376, 95% CI: 2.305–8.310, *p* < 0.001).

### 2.5. Multivariable Analysis of Independent Predictors of Antimicrobial Resistance

In the multivariable GEE model adjusted for patient clustering, several factors remained independently associated with ciprofloxacin antimicrobial resistance at the isolate level (Table 8).

Increasing age was independently associated with higher odds of antimicrobial resistance, with an adjusted odds ratio (aOR) of 1.02 per 1-year increase (95% CI: 1.009–1.024, *p* < 0.001). Antimicrobial resistance remained more frequent among isolates from male patients, with an aOR of 1.39 (95% CI: 1.042–1.844, *p* = 0.025) compared with isolates from female patients. No independent association was observed between antimicrobial resistance and environment of origin. Isolates from urban settings had an aOR of 1.02 (95% CI: 0.671–1.545, *p* = 0.933) compared with rural isolates.

After adjustment, pathogen-specific effects persisted for several organisms. Compared with *E. coli*, antimicrobial resistance was significantly more frequent in *P. mirabilis* (aOR 8.16, 95% CI: 4.079–16.333, *p* < 0.001) and *P. aeruginosa* (aOR 16.7, 95% CI: 5.229–53.349, *p* < 0.001). Isolates grouped as other pathogens also demonstrated higher odds of antimicrobial resistance compared with *E. coli* (aOR 2.06, 95% CI: 1.305–3.245, *p* < 0.001). In contrast, the association with *K. pneumoniae* did not remain statistically significant after adjustment (aOR 1.37, 95% CI: 0.902–2.082, *p* = 0.140).

ESBL production showed the strongest independent association with ciprofloxacin antimicrobial resistance. ESBL-positive isolates had an aOR of 9.83 (95% CI: 6.981–13.832, *p* < 0.001) compared with ESBL-negative isolates.

In the multivariable GEE model adjusted for patient clustering, fewer predictors remained independently associated with antimicrobial resistance to trimethoprim/sulfamethoxazole compared with ciprofloxacin (Table 9).

Age was not independently associated with antimicrobial resistance after adjustment (aOR 1.01 per 1-year increase, 95% CI: 1.000–1.014, *p* = 0.060). Similarly, no independent association was observed for sex, with isolates from male patients showing an aOR of 1.25 (95% CI: 0.950–1.634, *p* = 0.112) compared with female patients. Environment of origin was not associated with antimicrobial resistance. Isolates from urban settings had an aOR of 1.07 (95% CI: 0.715–1.596, *p* = 0.746) compared with rural isolates.

Pathogen-specific effects differed after adjustment. Compared with *E. coli*, *P. mirabilis* remained strongly associated with antimicrobial resistance, with an aOR of 12.2 (95% CI: 5.434–27.287, *p* < 0.001). The association with *K. pneumoniae* approached but did not reach statistical significance (aOR 1.40, 95% CI: 0.995–1.978, *p* = 0.053), while isolates grouped as other pathogens showed no independent association (aOR 0.813, 95% CI: 0.492–1.341, *p* = 0.417).

ESBL production remained an independent predictor of trimethoprim/sulfamethoxazole antimicrobial resistance. ESBL-positive isolates had an aOR of 2.89 (95% CI: 2.122–3.928, *p* < 0.001) compared with ESBL-negative isolates. This model contrasts with the ciprofloxacin model: fewer demographic effects persist, while pathogen identity (Proteus) and ESBL status dominate.

In the multivariable GEE model adjusted for patient clustering, several factors remained independently associated with antimicrobial resistance to amoxicillin/clavulanic acid (Table 10).

Age was not independently associated with antimicrobial resistance (aOR 0.996 per 1-year increase, 95% CI: 0.987–1.005, *p* = 0.407). Antimicrobial resistance remained more frequent among isolates from male patients, with an aOR of 1.50 (95% CI: 1.063–2.105, *p* = 0.021) compared with isolates from female patients. No independent association was observed between antimicrobial resistance and environment of origin. Isolates from urban settings had an aOR of 1.20 (95% CI: 0.709–2.027, *p* = 0.498) compared with rural isolates.

Pathogen identity remained a strong independent determinant of antimicrobial resistance. Compared with *E. coli*, antimicrobial resistance was significantly higher in *K. pneumoniae* (aOR 3.15, 95% CI: 2.073–4.799, *p* < 0.001), *P. mirabilis* (aOR 5.70, 95% CI: 2.625–12.393, *p* < 0.001), and isolates grouped as other pathogens, which demonstrated markedly increased odds of antimicrobial resistance (aOR 28.4, 95% CI: 16.281–49.565, *p* < 0.001).

ESBL production was independently associated with amoxicillin/clavulanic acid antimicrobial resistance. ESBL-positive isolates had an aOR of 3.22 (95% CI: 2.158–4.801, *p* < 0.001) compared with ESBL-negative isolates. This model further reinforces pathogen-specific effects and ESBL status as dominant drivers of antimicrobial resistance, while demographic effects are limited to sex.

In the multivariable GEE model adjusted for patient clustering, antimicrobial resistance to cefotaxime was predominantly driven by pathogen-related factors and ESBL production (Table 11).

Age was not independently associated with cefotaxime antimicrobial resistance after adjustment (aOR 1.01 per 1-year increase, 95% CI: 0.998–1.028, *p* = 0.091). Antimicrobial resistance was more frequent among isolates from male patients, with an aOR of 1.65 (95% CI: 1.017–2.666, *p* = 0.043) compared with isolates from female patients. No independent association was observed between antimicrobial resistance and environment of origin. Isolates from urban settings had an aOR of 1.41 (95% CI: 0.705–2.812, *p* = 0.333) compared with rural isolates.

Pathogen identity showed very strong independent associations with cefotaxime antimicrobial resistance. Compared with *E. coli*, antimicrobial resistance was markedly higher in *K. pneumoniae* (aOR 57.3, 95% CI: 16.948–193.653, *p* < 0.001), *P. mirabilis* (aOR 162, 95% CI: 50.334–522.009, *p* < 0.001), and isolates grouped as other pathogens (aOR 163, 95% CI: 55.897–474.974, *p* < 0.001).

ESBL production demonstrated the strongest independent association with cefotaxime antimicrobial resistance. ESBL-positive isolates had an aOR of 1337 (95% CI: 406.387–4395.937, *p* < 0.001) compared with ESBL-negative isolates.

This model clearly establishes ESBL status and pathogen identity as the dominant determinants of third-generation cephalosporin antimicrobial resistance, with minimal contribution from demographic or environmental factors.

**Figure 3:**
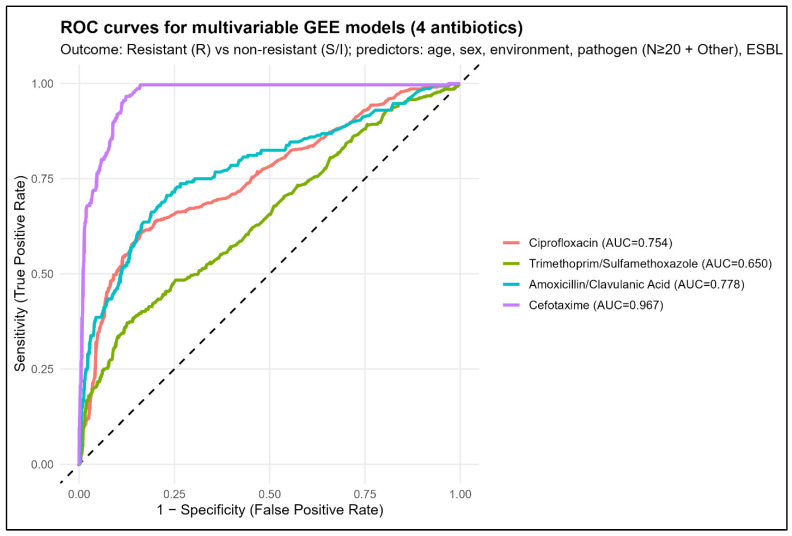
Receiver operating characteristic (ROC) curves for multivariable GEE models predicting antimicrobial resistance (R vs. S/I) for four antimicrobials.

The discriminative performance of the multivariable GEE models varied substantially across antimicrobials. The model predicting cefotaxime antimicrobial resistance demonstrated excellent discrimination, with an area under the curve (AUC) of 0.967, indicating a very high ability to distinguish resistant from non-resistant isolates. Good discrimination was observed for the amoxicillin/clavulanic acid model, which achieved an AUC of 0.778, followed by the ciprofloxacin model with an AUC of 0.754, both indicating acceptable to good predictive performance. In contrast, the model predicting trimethoprim/sulfamethoxazole antimicrobial resistance showed modest discrimination, with an AUC of 0.650, suggesting limited predictive accuracy compared with the other antimicrobials. Overall, model performance was highest for antimicrobials whose antimicrobial resistance was strongly driven by pathogen identity and ESBL status, particularly cefotaxime, whereas discrimination was lower for trimethoprim/sulfamethoxazole. The AUC of the cefotaxime model was significantly higher than that of the ciprofloxacin model (DeLong test, *p* < 0.001). The results are presented in Figure 3.

### 2.6. Supplementary MIC Analyses of Ciprofloxacin and Meropenem

MIC data were available for 434 isolates tested against ciprofloxacin and 400 isolates tested against meropenem. For ciprofloxacin, the median MIC was 0.25 mg/L, with a wide interquartile range (0.25–4.00 mg/L), indicating substantial variability in susceptibility across isolates. In contrast, meropenem exhibited a median MIC of 0.25 mg/L with a narrow interquartile range (0.25–0.25 mg/L), reflecting uniformly low MIC values among tested isolates.

For ciprofloxacin, MIC values differed significantly by sex (Table 12). Among isolates from female patients (n = 271), the median MIC was 0.25 mg/L (IQR: 0.25–4.00 mg/L), whereas isolates from male patients (n = 163) had a higher median MIC of 0.50 mg/L (IQR: 0.25–4.00 mg/L). This difference was statistically significant (*p* < 0.001). For meropenem, the median MIC was 0.25 mg/L in both female (n = 259) and male (n = 141) patients, with identical interquartile ranges (0.25–0.25 mg/L). Despite identical medians, a statistically significant difference in MIC distribution was observed between sexes (*p* = 0.020).

For ciprofloxacin, MIC values did not differ by environment (Table 13). Both rural (n = 44) and urban (n = 390) isolates had a median MIC of 0.25 mg/L with identical interquartile ranges (0.25–4.00 mg/L), and no statistically significant difference in MIC distribution was observed (*p* = 0.937). For meropenem, the median MIC was 0.25 mg/L in both rural (n = 41) and urban (n = 359) isolates, with identical interquartile ranges (0.25–0.25 mg/L). However, a statistically significant difference in MIC distribution between environments was observed (*p* < 0.001).

For ciprofloxacin, MIC values differed significantly among major pathogens (Kruskal–Wallis test, *p* < 0.001, Table 14). *E. coli* isolates (n = 294) had a median MIC of 0.25 mg/L (IQR: 0.25–2.00 mg/L), while *K. pneumoniae* (n = 52) showed a slightly higher median MIC of 0.375 mg/L (IQR: 0.25–4.00 mg/L). In contrast, substantially higher median MICs were observed for *E. faecalis* (n = 22; median 4.50 mg/L, IQR: 1.00–8.00 mg/L) and Serratia marcescens (n = 12; median 4.00 mg/L, IQR: 4.00–4.00 mg/L). For meropenem, MIC values also differed significantly across pathogens (Kruskal–Wallis test, *p* < 0.001). However, all major pathogens showed uniformly low MICs, with median values of 0.25 mg/L and identical interquartile ranges (0.25–0.25 mg/L) for *E. coli* (n = 294), *K. pneumoniae* (n = 52), and Serratia marcescens (n = 12).

For ciprofloxacin, MIC values differed significantly according to ESBL status (Table 15). Among ESBL-negative isolates (n = 364), the median MIC was 0.25 mg/L (IQR: 0.25–2.00 mg/L), whereas ESBL-positive isolates (n = 70) exhibited a markedly higher median MIC of 4.00 mg/L (IQR: 1.25–4.00 mg/L). This difference in MIC distribution was statistically significant (*p* < 0.001).

For meropenem, the median MIC was 0.25 mg/L for both ESBL-negative (n = 330) and ESBL-positive (n = 70) isolates, with identical interquartile ranges (0.25–0.25 mg/L). No statistically significant difference in MIC distribution was observed by ESBL status (*p* = 0.106).

## 3. Discussion

In this isolate-level study spanning six years, we provide a comprehensive analysis of antimicrobial resistance patterns among uropathogens isolated from patients with lower urinary tract infections and identify independent predictors of antimicrobial resistance using multivariable models accounting for patient clustering. Several important findings emerge from this analysis. First, antimicrobial resistance was highly heterogeneous across pathogens and antimicrobials, with marked differences between species. Second, ESBL production emerged as the most consistent and powerful independent determinant of antimicrobial resistance, not only to beta-lactam antimicrobials but also to non–beta-lactam agents such as fluoroquinolones and trimethoprim/sulfamethoxazole. Third, demographic factors played a secondary and antimicrobial-specific role, while environment of origin showed no consistent association with antimicrobial resistance [1,3,10].

The distribution of uropathogens in this cohort was consistent with previous reports, with *E. coli* accounting for approximately two-thirds of isolates, followed by *K. pneumoniae* and *E. faecalis*. Despite this stable pathogen distribution, susceptibility profiles varied substantially between organisms. *E. coli* retained high susceptibility to fosfomycin and nitrofurantoin, supporting their continued role as first-line oral agents for uncomplicated lower UTIs. In contrast, antimicrobial resistance to fluoroquinolones and trimethoprim/sulfamethoxazole remained substantial, limiting their empiric usefulness in many settings. These findings reinforce current concerns regarding the diminishing reliability of historically preferred oral agents for UTI treatment [4,5,11,20].

A central finding of this study is the dominant role of ESBL production in shaping antimicrobial resistance patterns. ESBL-positive isolates demonstrated markedly increased odds of antimicrobial resistance to ciprofloxacin, amoxicillin/clavulanic acid, and trimethoprim/sulfamethoxazole, and an exceptionally strong association with antimicrobial resistance to cefotaxime. The magnitude of these associations persisted after adjustment for age, sex, environment, and pathogen identity, highlighting ESBL status as an independent and robust marker of multidrug antimicrobial resistance. This observation aligns with growing evidence that ESBL production frequently coexists with antimicrobial resistance determinants affecting multiple antimicrobial classes, reflecting the plasmid-mediated clustering of antimicrobial resistance genes [7,8,21].

The exceptionally high adjusted odds ratios observed for cefotaxime antimicrobial resistance among ESBL-positive isolates underscore the central mechanistic link between ESBL enzymes and third-generation cephalosporin antimicrobial resistance. The excellent discriminative performance of the cefotaxime model further supports the notion that antimicrobial resistance to this antimicrobial is predominantly driven by ESBL production and pathogen identity, with minimal contribution from demographic or environmental factors. In contrast, models predicting antimicrobial resistance to trimethoprim/sulfamethoxazole demonstrated more modest discrimination, suggesting a more complex antimicrobial resistance landscape influenced by factors not fully captured in routine clinical datasets [8,19,22].

Pathogen-specific effects were another prominent feature of the analysis. *P. mirabilis* and *P. aeruginosa* exhibited markedly increased odds of antimicrobial resistance to ciprofloxacin and other antimicrobials, even after multivariable adjustment. These findings are clinically relevant, as both organisms are intrinsically less susceptible to several commonly used agents and are often associated with complicated infections or prior healthcare exposure. *K. pneumoniae* demonstrated antimicrobial-specific effects, remaining an independent predictor of antimicrobial resistance for certain antimicrobials but not others after adjustment, reflecting its heterogeneous antimicrobial resistance profile [16,23,24].

Demographic variables showed more limited and inconsistent associations with antimicrobial resistance. Male sex remained independently associated with antimicrobial resistance to several antimicrobials, including ciprofloxacin, amoxicillin/clavulanic acid, and cefotaxime. This finding may reflect differences in infection complexity, underlying urological conditions, or prior antimicrobial exposure, which were not directly measured in this study. Age showed a modest independent association with ciprofloxacin antimicrobial resistance but not with antimicrobial resistance to other antimicrobials after adjustment, suggesting that age-related effects may be antibiotic-specific rather than universal [18,25,26,27].

Notably, environment of origin (urban versus rural) was not independently associated with antimicrobial resistance or ESBL production across analyses. This finding suggests a relatively homogeneous distribution of resistant uropathogens across environments in this cohort and highlights the widespread dissemination of antimicrobial resistance beyond traditional healthcare-associated settings [6,17,28].

Supplementary analyses of minimum inhibitory concentrations provided additional insight into antimicrobial resistance gradients. ESBL-positive isolates demonstrated substantially higher ciprofloxacin MICs compared with ESBL-negative isolates, supporting the strong association observed in categorical antimicrobial resistance analyses. In contrast, meropenem MICs remained uniformly low across pathogens, ESBL status, sex, and environment, underscoring the preserved activity of carbapenems in this population. While these MIC analyses were exploratory and limited to a subset of isolates, they reinforce the main findings derived from susceptibility categorizations [19,29,30].

This study has several strengths. The large sample size, extended study period, and isolate-level analytical approach allowed for detailed characterization of antimicrobial resistance patterns and robust multivariable modeling. The use of generalized estimating equations accounted for within-patient correlation, reducing the risk of biased estimates. In addition, the inclusion of ESBL status and MIC data provided a more nuanced assessment of antimicrobial resistance mechanisms beyond categorical susceptibility results [12,14,15].

Several limitations should also be acknowledged. The retrospective design precluded the assessment of clinical outcomes, prior antimicrobial exposure, comorbidities, or healthcare contact, which may influence antimicrobial resistance patterns. The study did not include clinical outcome data such as treatment response or patient prognosis, as the analysis was based on microbiological laboratory records rather than clinical follow-up. Susceptibility testing was not uniform across all isolates and antimicrobials, reflecting real-world laboratory practice. The study was conducted at a single center, which may limit generalizability to other regions. Finally, MIC data were available only for selected antimicrobials and a subset of isolates and were therefore considered supportive rather than definitive [2,31,32].

## 4. Materials and Methods

### 4.1. Study Design and Setting

This retrospective observational study was conducted at Petroșani Emergency Hospital, Romania, and included data collected between 1 January 2019 and 31 December 2024. The study evaluated microbiological findings and antimicrobial susceptibility profiles among patients presenting with symptoms suggestive of lower urinary tract infection (UTI) and undergoing urine microbiological testing as part of routine clinical care.

### 4.2. Study Population

The study population consisted of consecutive patients who presented to Petroșani Emergency Hospital during the study period with clinical features compatible with lower UTI and for whom urine culture and antimicrobial susceptibility testing were performed.

Patients were included if they met all of the following criteria:

1. Presentation to Petroșani Emergency Hospital between 2019 and 2024;
2. Clinical suspicion of lower urinary tract infection (e.g., dysuria, urinary frequency, urgency, suprapubic discomfort), as documented in medical records;
3. Availability of urine culture results with pathogen identification;
4. Availability of antimicrobial susceptibility testing results for at least one antimicrobial.

The following records were excluded from the analysis:

1. Duplicate samples from the same patient and pathogen, retaining only one isolate per patient–pathogen combination;
2. Urine samples considered contaminated according to laboratory criteria (e.g., mixed growth of multiple organisms suggestive of improper collection);
3. Cultures reported as negative or with no bacterial growth;
4. Isolates lacking definitive susceptibility interpretation (S/I/R);
5. Records with missing key demographic data (age or sex);
6. Asymptomatic bacteriuria identified incidentally without clinical signs of lower UTI;
7. Samples collected from patients with indwelling urinary catheters;
8. Samples collected in the context of upper urinary tract infection or pyelonephritis;
9. Pediatric patients;
10. Pregnant patients.

### 4.3. Data Sources and Variables

Data were extracted retrospectively from the laboratory information system and corresponding electronic medical records. The following variables were collected: demographic variables (age–years, sex); environment of origin (urban or rural), defined according to the patient’s registered residence; microbiological variables (pathogen identification, antimicrobial susceptibility results—susceptible, intermediate, resistant, ESBL status for Gram-negative isolates, and MIC values when available); testing variables (antimicrobials tested per isolate and completeness of susceptibility panels).

### 4.4. Urine Sample Collection and Processing

Urine samples were collected under routine clinical conditions, predominantly as midstream clean-catch urine specimens following standard patient instructions. Samples were transported to the microbiology laboratory and processed within 2–4 h of collection or stored at 4 °C until processing, according to routine laboratory protocols.

### 4.5. Microbiological Culture and Identification

Urine specimens were cultured using standard bacteriological techniques. Samples were inoculated using calibrated loops onto blood agar and MacConkey agar and incubated at 35–37 °C for 18–24 h. Significant bacteriuria was determined according to laboratory criteria based on colony counts and clinical context.

Bacterial identification was performed using an automated identification system (VITEK 2 Compact, bioMérieux, Marcy-l’Étoile, France). When required according to routine laboratory practice, identification was supplemented by conventional biochemical tests.

### 4.6. Definition of Isolates and Units of Analysis

To avoid repeated measurements from the same patient, predefined analytical units were used: patient-level analysis (one record per patient, used for demographic descriptions); isolate-level analysis (one isolate per unique patient–pathogen combination); isolate–antimicrobial test-level analysis (each susceptibility result for a specific antimicrobial constituted one observation). Duplicate isolates from the same patient and pathogen were excluded during data preprocessing, and repeated infections from the same patient were not counted separately. Therefore, only one isolate per patient was included in the final dataset.

### 4.7. Antimicrobial Susceptibility Testing

Antimicrobial susceptibility testing (AST) was performed as part of routine laboratory practice using an automated system (VITEK 2 Compact, bioMérieux) and/or the disk diffusion method, depending on the organism and laboratory protocol. For disk diffusion testing, bacterial inoculum suspensions were prepared from fresh cultures and adjusted to a 0.5 McFarland turbidity standard, in accordance with standard microbiological procedures.

Antimicrobial susceptibility results were interpreted according to the European Committee on Antimicrobial Susceptibility Testing (EUCAST) guidelines applicable during the study period (2019–2024). Susceptibility categories were reported as susceptible (S), susceptible with increased exposure (I), or resistant (R) according to the EUCAST breakpoints in use at the time of testing. No retrospective reinterpretation of susceptibility results was performed.

Due to variations in routine testing practices over time, not all isolates were tested against all antimicrobial agents. EUCAST interpretative criteria were applied for species-specific susceptibility interpretation, including recognition of intrinsic resistance mechanisms where applicable.

### 4.8. ESBL Detection

Extended-spectrum beta-lactamase (ESBL) production among Gram-negative isolates was determined using routine phenotypic methods implemented in the clinical microbiology laboratory. Initial screening was performed using the automated susceptibility testing system (VITEK 2 Compact, bioMérieux). When required, ESBL production was confirmed using phenotypic confirmatory testing based on EUCAST recommendations, including the combination disk method with third-generation cephalosporins (e.g., cefotaxime or ceftazidime) with and without clavulanic acid. Gram-negative isolates were classified as ESBL-positive or ESBL-negative for analytical purposes.

### 4.9. Minimum Inhibitory Concentration (MIC) Data

MIC values were available for a subset of isolates, primarily for ciprofloxacin and meropenem, and were obtained using the automated susceptibility testing system. MIC values were analyzed as supportive data to explore antimicrobial resistance gradients and were not available for all isolates.

### 4.10. Outcome Definitions

For antimicrobial resistance modeling, outcomes were defined at the isolate level as resistant (R) versus non-resistant (susceptible or intermediate, S/I) for selected antimicrobials, including ciprofloxacin, trimethoprim/sulfamethoxazole, amoxicillin/clavulanic acid, and cefotaxime.

### 4.11. Statistical Analysis

A comprehensive statistical analysis was conducted to characterize antimicrobial susceptibility patterns and to identify factors independently associated with antimicrobial resistance among uropathogens isolated between 2019 and 2024. The primary outcomes were antimicrobial resistance phenotypes, defined at the isolate level as resistant (R) versus susceptible or intermediate (S/I), according to routine susceptibility testing.

The analysis had four main objectives: (1) to describe patient characteristics and pathogen distribution; (2) to summarize global and pathogen-specific antimicrobial susceptibility profiles; (3) to evaluate the prevalence and distribution of extended-spectrum beta-lactamase (ESBL) production among Gram-negative isolates; and (4) to identify independent predictors of antimicrobial resistance to selected antimicrobials using multivariable modeling.

Continuous variables were summarized using medians and interquartile ranges (IQRs), while categorical variables were summarized as absolute frequencies and percentages. Normality of continuous variables was assessed using the Shapiro–Wilk test; due to non-normal distributions, non-parametric summaries were used throughout.

Patient-level analyses included one record per patient, while isolate-level analyses were defined as unique patient–pathogen combinations, with one isolate per patient included for isolate-level comparisons. Analyses of antimicrobial susceptibility at the isolate–antimicrobial test level were restricted to tests with available interpretive categories (susceptible, intermediate, resistant).

Comparisons of continuous variables between two groups were performed using the Mann–Whitney U test, while comparisons across more than two groups used the Kruskal–Wallis test. Categorical variables were compared using Pearson’s Chi-squared test. When expected cell counts were small, Monte Carlo simulation was applied to obtain robust *p*-values.

Analyses involving pathogens were restricted to organisms with at least 20 isolates for main comparisons; pathogens with smaller counts were grouped as “other” or presented in Supplementary Materials.

Global antimicrobial susceptibility profiles were summarized for antimicrobials with sufficient testing frequency, while complete susceptibility data were provided in Supplementary tables. ESBL production was evaluated among Gram-negative isolates, and its distribution was examined overall, by pathogen, and by environment. Associations between ESBL status and categorical variables were assessed using Chi-squared tests, with Monte Carlo simulation where appropriate.

Univariate analyses were conducted to explore associations between selected predictors and antimicrobial resistance to ciprofloxacin, trimethoprim/sulfamethoxazole, amoxicillin/clavulanic acid, and cefotaxime. Predictors included age, sex, environment (urban vs. rural), pathogen category, and ESBL status (Gram-negative isolates only). Unadjusted odds ratios (ORs) with 95% confidence intervals (CIs) were estimated using Chi-squared tests or univariate logistic regression, as appropriate.

To identify independent predictors of antimicrobial resistance while accounting for within-patient correlation, multivariable generalized estimating equation (GEE) logistic regression models were constructed for each antimicrobial. Models included age (continuous), sex, environment, pathogen category, and ESBL status, with clustering by patient identifier. Adjusted odds ratios (aORs) with 95% CIs were reported.

Model discrimination was evaluated using receiver operating characteristic (ROC) curves, and predictive performance was summarized using the area under the curve (AUC).

Minimum inhibitory concentration (MIC) data were analyzed for ciprofloxacin and meropenem among isolates with available MIC values. MICs were summarized using medians and IQRs and compared across sex, environment, pathogen categories, and ESBL status using the Mann–Whitney U test or Kruskal–Wallis test, as appropriate. These analyses were considered supportive and were presented in Supplementary Materials.

All statistical tests were two-sided. Statistical significance was defined as *p* < 0.05, with *p*-values <0.001 reported accordingly. Data analysis was performed using R (version 4.3.0) and RStudio (version 2023.06.0+421).

## 5. Conclusions

In this isolate-level study spanning 2019–2024, antimicrobial resistance among uropathogens was heterogeneous and strongly influenced by pathogen identity and ESBL production. Across multiple antimicrobials, ESBL status emerged as the most consistent and powerful independent predictor of antimicrobial resistance, particularly for ciprofloxacin and third-generation cephalosporins.

Multivariable GEE analyses demonstrated that antimicrobial resistance to ciprofloxacin, trimethoprim/sulfamethoxazole, amoxicillin/clavulanic acid, and cefotaxime was not uniformly distributed across pathogens. *P. mirabilis* and *P. aeruginosa* showed markedly increased odds of antimicrobial resistance for selected antimicrobials, while *K. pneumoniae* exhibited antimicrobial-specific effects that persisted after adjustment in some models. Demographic factors played a more limited role; male sex was independently associated with antimicrobial resistance for certain antimicrobials, whereas environment of origin showed no consistent association.

Models predicting cefotaxime antimicrobial resistance demonstrated excellent discriminative performance, reflecting the dominant contribution of ESBL production and pathogen identity. In contrast, models for trimethoprim/sulfamethoxazole showed more modest discrimination, indicating a more complex and less predictable antimicrobial resistance profile. Minimum inhibitory concentration analyses further supported these findings, particularly by demonstrating substantially higher ciprofloxacin MICs among ESBL-positive isolates, while meropenem MICs remained uniformly low irrespective of ESBL status.

Overall, these findings underscore the central role of ESBL production and pathogen-specific characteristics in shaping antimicrobial resistance among uropathogens. The results support the need for pathogen-informed and antimicrobial resistance-aware approaches to antimicrobial selection, while highlighting the value of multivariable modeling in disentangling independent contributors to antimicrobial resistance.

## Figures and Tables

**Table 1:** Baseline demographic characteristics of patients with urinary tract infections (patient-level analysis).

Characteristic	Total (N = 1470)
Age (years), median (IQR)	66 (54–75)
Sex, n (%)	
Female	964 (65.6%)
Male	506 (34.4%)
Environment, n (%)	
Rural	135 (9.2%)
Urban	1335 (90.8%)

**Table 2:** Global antimicrobial susceptibility profile of selected clinically relevant antimicrobials (isolate–antimicrobial test-level analysis).

Antimicrobial	n Tested	Susceptible, n (%)	Susceptible, Increased Exposure, n (%)	Resistant, n (%)
Ciprofloxacin	1443	899 (62.3%)	33 (2.3%)	511 (35.4%)
Gentamicin	1317	1077 (81.8%)	6 (0.5%)	234 (17.8%)
Ceftazidime	1295	1058 (81.7%)	9 (0.7%)	228 (17.6%)
Meropenem	1292	1245 (96.4%)	4 (0.3%)	43 (3.3%)
Amikacin	1291	1189 (92.1%)	58 (4.5%)	44 (3.4%)
Cefotaxime	1284	981 (76.4%)	3 (0.2%)	300 (23.4%)
Amoxicillin/Clavulanic Acid	1280	894 (69.8%)	153 (12.0%)	233 (18.2%)
Cefepime	1278	1087 (85.1%)	8 (0.6%)	183 (14.3%)
Imipenem	1276	1181 (92.6%)	31 (2.4%)	64 (5.0%)
Trimethoprim/Sulfamethoxazole	1275	871 (68.3%)	0 (0.0%)	404 (31.7%)
Ertapenem	1242	1226 (98.7%)	3 (0.2%)	13 (1.0%)
Nitrofurantoin	1233	955 (77.5%)	131 (10.6%)	147 (11.9%)
Fosfomycin	1197	1170 (97.7%)	1 (0.1%)	26 (2.2%)
Cefuroxime	899	643 (71.5%)	8 (0.9%)	248 (27.6%)
Norfloxacin	615	67 (10.9%)	0 (0.0%)	548 (89.1%)
Ceftriaxone	562	469 (83.5%)	0 (0.0%)	93 (16.5%)
Levofloxacin	223	118 (52.9%)	11 (4.9%)	94 (42.2%)

**Table 3:** Distribution of ESBL-producing Gram-negative isolates by environment.

Environment	Total GN Isolates, n	ESBL-Positive, n (%)	ESBL-Negative, n (%)
Urban	1162	197 (17.0)	965 (83.0)
Rural	125	26 (20.8)	99 (79.2)

**Table 4:** Univariate associations with ciprofloxacin antimicrobial resistance (R vs. S/I) at isolate level (pathogens with N ≥ 20).

Predictor	Comparison (vs. Reference)	Test	Unadjusted OR (95% CI)	*p*-Value
Sex	Male vs. Female	Chi-square	1.77 (1.404–2.231)	<0.001
Environment	Rural vs. Urban	Chi-square	1.15 (0.781–1.688)	0.449
ESBL status (Gram-negatives only)	Positive vs. Negative	Chi-square	8.33 (5.954–11.778)	<0.001
Pathogen	*E. faecalis* vs. *E. coli*	Univariate logistic (pathogens N ≥ 20)	3.31 (2.187–5.003)	<0.001
Pathogen	*K. pneumoniae* vs. *E. coli*	Univariate logistic (pathogens N ≥ 20)	2.12 (1.502–2.989)	<0.001
Pathogen	*P. mirabilis* vs. *E. coli*	Univariate logistic (pathogens N ≥ 20)	4.95 (2.557–9.581)	<0.001
Pathogen	*P. aeruginosa* vs. *E. coli*	Univariate logistic (pathogens N ≥ 20)	11.5 (3.873–34.431)	<0.001

**Table 5:** Univariate associations with trimethoprim/sulfamethoxazole antimicrobial resistance (R vs. S/I) at isolate level (pathogens N ≥ 20).

Predictor	Comparison (vs. Reference)	Test	Unadjusted OR (95% CI)	*p*-Value
Sex	Male vs. Female	Chi-square	1.293 (0.999–1.672)	0.043
Environment	Rural vs. Urban	Chi-square	0.996 (0.650–1.506)	0.996
ESBL status (Gram-negatives only)	Positive vs. Negative	Chi-square	2.882 (2.116–3.929)	<0.001
Pathogen	*K. pneumoniae* vs. *E. coli*	Univariate logistic (pathogens N ≥ 20)	1.789 (1.266–2.528)	<0.001
Pathogen	*P. mirabilis* vs. *E. coli*	Univariate logistic (pathogens N ≥ 20)	9.912 (4.521–21.730)	<0.001

**Table 6:** Univariate associations with amoxicillin/clavulanic acid antimicrobial resistance (R vs. S/I) at isolate level (pathogens N ≥ 20).

Predictor	Comparison (vs. Reference)	Test	Unadjusted OR (95% CI)	*p*-Value
Sex	Male vs. Female	Chi-square	2.359 (1.739–3.199)	<0.001
Environment	Rural vs. Urban	Chi-square	1.163 (0.692–1.892)	0.531
ESBL status (Gram-negatives only)	Positive vs. Negative	Chi-square	2.230 (1.571–3.146)	<0.001
Pathogen	*K. pneumoniae* vs. *E. coli*	Univariate logistic (pathogens N ≥ 20)	3.929 (2.652–5.820)	<0.001
Pathogen	*P. mirabilis* vs. *E. coli*	Univariate logistic (pathogens N ≥ 20)	4.404 (2.065–9.394)	<0.001

**Table 7:** Univariate associations with cefotaxime antimicrobial resistance (R vs. S/I) at isolate level (pathogens N ≥ 20).

Predictor	Comparison (vs. Reference)	Test	Unadjusted OR (95% CI)	*p*-Value
Sex	Male vs. Female	Chi-square	2.215 (1.679–2.923)	<0.001
Environment	Rural vs. Urban	Chi-square	1.275 (0.808–1.975)	0.259
ESBL status (Gram-negatives only)	Positive vs. Negative	Chi-square	91.142 (55.338–156.710)	<0.001
Pathogen	*K. pneumoniae* vs. *E. coli*	Univariate logistic (pathogens N ≥ 20)	5.810 (4.058–8.317)	<0.001
Pathogen	*P. mirabilis* vs. *E. coli*	Univariate logistic (pathogens N ≥ 20)	4.376 (2.305–8.310)	<0.001

**Table 8:** Multivariable analysis of ciprofloxacin antimicrobial resistance (R vs. S/I) at isolate level (GEE, clustered by patient).

Predictor	Comparison (vs. Reference)	Adjusted OR (95% CI)	*p*-Value
Age	per 1-year increase	1.02 (1.009–1.024)	<0.001
Sex	Male vs. Female	1.39 (1.042–1.844)	0.025
Environment	Urban vs. Rural	1.02 (0.671–1.545)	0.933
Pathogen	*K. pneumoniae* vs. *E. coli*	1.37 (0.902–2.082)	0.140
Pathogen	Other vs. *E. coli*	2.06 (1.305–3.245)	0.002
Pathogen	*P. mirabilis* vs. *E. coli*	8.16 (4.079–16.333)	<0.001
Pathogen	*P. aeruginosa* vs. *E. coli*	16.7 (5.229–53.349)	<0.001
ESBL status	Positive vs. Negative	9.83 (6.981–13.832)	<0.001

**Table 9:** Multivariable analysis of trimethoprim/sulfamethoxazole antimicrobial resistance (R vs. S/I) at isolate level (GEE, clustered by patient).

Predictor	Comparison (vs. Reference)	Adjusted OR (95% CI)	*p*-Value
Age	per 1-year increase	1.01 (1.000–1.014)	0.060
Sex	Male vs. Female	1.25 (0.950–1.634)	0.112
Environment	Urban vs. Rural	1.07 (0.715–1.596)	0.746
Pathogen	*K. pneumoniae* vs. *E. coli*	1.40 (0.995–1.978)	0.053
Pathogen	Other vs. *E. coli*	0.813 (0.492–1.341)	0.417
Pathogen	*P. mirabilis* vs. *E. coli*	12.2 (5.434–27.287)	<0.001
ESBL status	Positive vs. Negative	2.89 (2.122–3.928)	<0.001

**Table 10:** Multivariable analysis of amoxicillin/clavulanic acid antimicrobial resistance (R vs. S/I) at isolate level (GEE, clustered by patient).

Predictor	Comparison (vs. Reference)	Adjusted OR (95% CI)	*p*-Value
Age	per 1-year increase	0.996 (0.987–1.005)	0.407
Sex	Male vs. Female	1.50 (1.063–2.105)	0.021
Environment	Urban vs. Rural	1.20 (0.709–2.027)	0.498
Pathogen	*K. pneumoniae* vs. *E. coli*	3.15 (2.073–4.799)	<0.001
Pathogen	Other vs. *E. coli*	28.4 (16.281–49.565)	<0.001
Pathogen	*P. mirabilis* vs. *E. coli*	5.70 (2.625–12.393)	<0.001
ESBL status	Positive vs. Negative	3.22 (2.158–4.801)	<0.001

**Table 11:** Multivariable analysis of cefotaxime antimicrobial resistance (R vs. S/I) at isolate level (GEE, clustered by patient).

Predictor	Comparison (vs. Reference)	Adjusted OR (95% CI)	*p*-Value
Age	per 1-year increase	1.01 (0.998–1.028)	0.091
Sex	Male vs. Female	1.65 (1.017–2.666)	0.043
Environment	Urban vs. Rural	1.41 (0.705–2.812)	0.333
Pathogen	*K. pneumoniae* vs. *E. coli*	57.3 (16.948–193.653)	<0.001
Pathogen	Other vs. *E. coli*	163 (55.897–474.974)	<0.001
Pathogen	*P. mirabilis* vs. *E. coli*	162 (50.334–522.009)	<0.001
ESBL status	Positive vs. Negative	1337 (406.387–4395.937)	<0.001

**Table 12:** Minimum inhibitory concentration (MIC) distribution by sex for ciprofloxacin and meropenem (isolate-level analysis).

Antimicrobial	Sex	n	Median MIC	IQR (Q1–Q3)	*p*-Value
Ciprofloxacin	Female	271	0.25	0.25–4.00	<0.001
	Male	163	0.50	0.25–4.00	
Meropenem	Female	259	0.25	0.25–0.25	0.020
	Male	141	0.25	0.25–0.25	

**Table 13:** Minimum inhibitory concentration (MIC) distribution by environment for ciprofloxacin and meropenem (isolate-level analysis).

Antimicrobial	Environment	n	Median MIC	IQR (Q1–Q3)	*p*-Value
Ciprofloxacin	Rural	44	0.25	0.25–4.00	0.937
	Urban	390	0.25	0.25–4.00	
Meropenem	Rural	41	0.25	0.25–0.25	<0.001
	Urban	359	0.25	0.25–0.25	

**Table 14:** Minimum inhibitory concentration (MIC) distribution by major pathogens for ciprofloxacin and meropenem (isolate-level analysis).

Antimicrobial	Pathogen	n	Median MIC	IQR (Q1–Q3)
Ciprofloxacin	*E. coli*	294	0.25	0.25–2.00
	*K. pneumoniae*	52	0.375	0.25–4.00
	*E. faecalis*	22	4.50	1.00–8.00
	*Serratia marcescens*	12	4.00	4.00–4.00
Kruskal–Wallis *p*-value				<0.001
Meropenem	*E. coli*	294	0.25	0.25–0.25
	*K. pneumoniae*	52	0.25	0.25–0.25
	*Serratia marcescens*	12	0.25	0.25–0.25
Kruskal–Wallis *p*-value				<0.001

**Table 15:** Minimum inhibitory concentration (MIC) distribution by ESBL status for ciprofloxacin and meropenem (isolate-level analysis).

Antimicrobial	ESBL Status	n	Median MIC	IQR (Q1–Q3)	*p*-Value
Ciprofloxacin	Negative	364	0.25	0.25–2.00	<0.001
	Positive	70	4.00	1.25–4.00	
Meropenem	Negative	330	0.25	0.25–0.25	0.106
	Positive	70	0.25	0.25–0.25	

## Data Availability

The original contributions presented in this study are included in the article/Supplementary Materials. Further inquiries can be directed to the corresponding authors.

## Supplementary Materials

The following supporting information can be downloaded at: https://www.mdpi.com/article/10.3390/antibiotics15030323/s1, Supplementary Table S1. Distribution of uropathogens isolated from patients with urinary tract infections (isolate-level analysis), Supplementary Table S2. Global antimicrobial susceptibility profile by antimicrobial (isolate–antimicrobial test-level analysis), Supplementary Table S3. Antimicrobial susceptibility profile of *E. coli* isolates to selected clinically relevant antimicrobials (isolate-level analysis), Supplementary Table S4. Antimicrobial susceptibility profile for *K. pneumoniae* (isolate-level analysis), Supplementary Table S5. Antimicrobial susceptibility profile for *E. faecalis* (isolate-level analysis), Supplementary Table S6. Distribution of ESBL-producing Gram-negative isolates by pathogen.

## Abbreviations

The following abbreviations are used in this manuscript:

AST	Antimicrobial Susceptibility Testing
AUC	Area Under the Curve
CI	Confidence Interval
ESBL	Extended-Spectrum Beta-Lactamase
EUCAST	European Committee on Antimicrobial Susceptibility Testing
GEE	Generalized Estimating Equation
GN	Gram-Negative
IQR	Interquartile Range
MIC	Minimum Inhibitory Concentration
OR	Odds Ratio
aOR	Adjusted Odds Ratio
ROC	Receiver Operating Characteristic
UTI	Urinary Tract Infection
