# Supplementary material for: Antimicrobial Resistance and ESBL-Associated Predictors Among Uropathogens: A 2019–2024 Isolate-Level Study
Source: Antibiotics (Basel). 2026 Mar 23;15(3):323. doi: 10.3390/antibiotics15030323 (PMC13024478; doi:10.3390/antibiotics15030323)
Supplement: Supplementary file 1 [file antibiotics-15-00323-s001.zip › antibiotics-4196215-supplementary.pdf]

Supplementary

Supplementary Table S1. Distribution of uropathogens isolated from patients with urinary tract infections (isolate-level analysis)

Pathogen	n	%
Escherichia coli	970	66.0
Klebsiella pneumoniae	157	10.7
Enterococcus faecalis	109	7.4
Proteus mirabilis	41	2.8
Streptococcus agalactiae	28	1.9
Pseudomonas aeruginosa	22	1.5
Serratia marcescens	19	1.3
Enterobacter cloacae complex	17	1.2
Enterococcus faecium	16	1.1
Citrobacter freundii	13	0.9
Staphylococcus aureus	11	0.7
Klebsiella oxytoca	10	0.7
Enterococcus faecium (VRE)	5	0.3
Staphylococcus epidermidis	5	0.3
Acinetobacter baumannii complex	4	0.3
Enterobacter aerogenes	4	0.3
Morganella morganii ssp morganii	4	0.3
Raoultella planticola	4	0.3
Staphylococcus saprophyticus	4	0.3
Citrobacter koseri	3	0.2
Enterobacter asburiae	3	0.2
Enterobacter cancerogenus	2	0.1
Myroides spp	2	0.1
Providencia stuartii	2	0.1
Alcaligenes faecalis ssp faecalis	1	0.1
Enterococcus casseliflavus	1	0.1
Morganella morganii ssp sibonii	1	0.1
Pantoea agglomerans	1	0.1
Proteus hauseri	1	0.1
Raoultella ornithinolytica	1	0.1
Salmonella group	1	0.1

Pathogen	n	%
Salmonella ser.Typhi	1	0.1
Serratia fonticola	1	0.1
Staphylococcus capitis	1	0.1
Staphylococcus haemolyticus	1	0.1
Staphylococcus hominis ssp hominis	1	0.1
Stenotrophomonas maltophilia	1	0.1
Streptococcus alactolyticus	1	0.1
Vibrio alginolyticus	1	0.1

Supplementary Table S2. Global antimicrobial susceptibility profile by antibiotic (isolate–antibiotic test-level analysis)

Antibiotic	n tested	Susceptible, n (%)	Susceptible, Increased Exposure, n (%)	Resistant, n (%)
Ciprofloxacin	1443	899 (62.3%)	33 (2.3%)	511 (35.4%)
Ampicillin	1362	559 (41.0%)	10 (0.7%)	793 (58.2%)
Gentamicin	1317	1077 (81.8%)	6 (0.5%)	234 (17.8%)
Ceftazidime	1295	1058 (81.7%)	9 (0.7%)	228 (17.6%)
Meropenem	1292	1245 (96.4%)	4 (0.3%)	43 (3.3%)
Amikacin	1291	1189 (92.1%)	58 (4.5%)	44 (3.4%)
Cefotaxime	1284	981 (76.4%)	3 (0.2%)	300 (23.4%)
Amoxicillin/Clavulanic Acid	1280	894 (69.8%)	153 (12.0%)	233 (18.2%)
Cefepime	1278	1087 (85.1%)	8 (0.6%)	183 (14.3%)
Imipenem	1276	1181 (92.6%)	31 (2.4%)	64 (5.0%)
Trimethoprim/Sulfamethoxazole	1275	871 (68.3%)	0 (0.0%)	404 (31.7%)
Piperacillin/Tazobactam	1263	1082 (85.7%)	48 (3.8%)	133 (10.5%)
Ertapenem	1242	1226 (98.7%)	3 (0.2%)	13 (1.0%)
Nitrofurantoin	1233	955 (77.5%)	131 (10.6%)	147 (11.9%)
Fosfomycin	1197	1170 (97.7%)	1 (0.1%)	26 (2.2%)
Cefuroxime	899	643 (71.5%)	8 (0.9%)	248 (27.6%)
Cefixime	895	687 (76.8%)	2 (0.2%)	206 (23.0%)
Norfloxacin	615	67 (10.9%)	0 (0.0%)	548 (89.1%)
Tobramycin	603	515 (85.4%)	4 (0.7%)	84 (13.9%)
Ceftriaxone	562	469 (83.5%)	0 (0.0%)	93 (16.5%)
Ceftazidime/Avibactam	545	545 (100.0%)	0 (0.0%)	0 (0.0%)
Ceftolozane/Tazobactam	431	431 (100.0%)	0 (0.0%)	0 (0.0%)
Tigecycline	394	355 (90.1%)	3 (0.8%)	36 (9.1%)

Antibiotic	n tested	Susceptible, n (%)	Susceptible, Increased Exposure, n (%)	Resistant, n (%)
Cefpodoxime	393	301 (76.6%)	0 (0.0%)	92 (23.4%)
Tetracycline	385	119 (30.9%)	2 (0.5%)	264 (68.6%)
Netilmicin	372	362 (97.3%)	0 (0.0%)	10 (2.7%)
Moxifloxacin	255	164 (64.3%)	3 (1.2%)	88 (34.5%)
Piperacillin	232	70 (30.2%)	4 (1.7%)	158 (68.1%)
Ticarcillin/Clavulanic Acid	231	145 (62.8%)	19 (8.2%)	67 (29.0%)
Cefaclor	226	131 (58.0%)	0 (0.0%)	95 (42.0%)
Levofloxacin	223	118 (52.9%)	11 (4.9%)	94 (42.2%)
Ofloxacin	216	116 (53.7%)	1 (0.5%)	99 (45.8%)
Ticarcillin	213	67 (31.5%)	0 (0.0%)	146 (68.5%)
Minocycline	209	127 (60.8%)	22 (10.5%)	60 (28.7%)
Aztreonam	208	163 (78.4%)	1 (0.5%)	44 (21.2%)
Cefoxitin	203	159 (78.3%)	9 (4.4%)	35 (17.2%)
Cefuroxime Axetil	203	116 (57.1%)	10 (4.9%)	77 (37.9%)
Nalidixic Acid	203	104 (51.2%)	0 (0.0%)	99 (48.8%)
Chloramphenicol	197	123 (62.4%)	31 (15.7%)	43 (21.8%)
Colistin	197	13 (6.6%)	150 (76.1%)	34 (17.3%)
Cefalotin	195	82 (42.1%)	26 (13.3%)	87 (44.6%)
Vancomycin	179	171 (95.5%)	0 (0.0%)	8 (4.5%)
Linezolid	174	168 (96.6%)	4 (2.3%)	2 (1.1%)
Teicoplanin	155	151 (97.4%)	0 (0.0%)	4 (2.6%)
Erythromycin	151	14 (9.3%)	1 (0.7%)	136 (90.1%)
Gentamicin High Level (synergy)	131	52 (39.7%)	0 (0.0%)	79 (60.3%)
Streptomycin High Level (synergy)	131	57 (43.5%)	0 (0.0%)	74 (56.5%)
Trimethoprim	110	61 (55.5%)	0 (0.0%)	49 (44.5%)
Clindamycin	52	18 (34.6%)	1 (1.9%)	33 (63.5%)
Cefoperazone/Sulbactam	50	50 (100.0%)	0 (0.0%)	0 (0.0%)
Azithromycin	37	6 (16.2%)	0 (0.0%)	31 (83.8%)
Benzylpenicillin	26	4 (15.4%)	0 (0.0%)	22 (84.6%)
Fusidic Acid	23	16 (69.6%)	0 (0.0%)	7 (30.4%)
Oxacillin	23	10 (43.5%)	0 (0.0%)	13 (56.5%)
Rifampicin	23	19 (82.6%)	0 (0.0%)	4 (17.4%)

Supplementary Table S3. Antimicrobial susceptibility profile of *Escherichia coli* isolates to selected clinically relevant antibiotics (isolate-level analysis)

Antibiotic	n tested	Susceptible, n (%)	Susceptible, Increased Exposure, n (%)	Resistant, n (%)
Fosfomycin	1072	1068 (99.6%)	0 (0.0%)	4 (0.4%)
Amoxicillin/Clavulanic Acid	997	763 (76.5%)	129 (12.9%)	105 (10.5%)
Amikacin	973	934 (96.0%)	34 (3.5%)	5 (0.5%)
Ceftazidime	973	879 (90.3%)	4 (0.4%)	90 (9.2%)
Ciprofloxacin	973	687 (70.6%)	14 (1.4%)	272 (28.0%)
Gentamicin	973	865 (88.9%)	2 (0.2%)	106 (10.9%)
Ampicillin	972	406 (41.8%)	8 (0.8%)	558 (57.4%)
Cefotaxime	972	824 (84.8%)	1 (0.1%)	147 (15.1%)
Meropenem	971	970 (99.9%)	1 (0.1%)	0 (0.0%)
Ertapenem	970	969 (99.9%)	0 (0.0%)	1 (0.1%)
Imipenem	970	969 (99.9%)	1 (0.1%)	0 (0.0%)
Piperacillin/Tazobactam	965	882 (91.4%)	29 (3.0%)	54 (5.6%)
Cefepime	963	895 (92.9%)	5 (0.5%)	63 (6.5%)
Trimethoprim/Sulfamethoxazole	949	671 (70.7%)	0 (0.0%)	278 (29.3%)
Nitrofurantoin	946	879 (92.9%)	56 (5.9%)	11 (1.2%)
Cefuroxime	724	588 (81.2%)	5 (0.7%)	131 (18.1%)
Cefixime	679	594 (87.5%)	1 (0.1%)	84 (12.4%)
Norfloxacin	506	57 (11.3%)	0 (0.0%)	449 (88.7%)
Ceftazidime/Avibactam	441	441 (100.0%)	0 (0.0%)	0 (0.0%)
Tobramycin	441	410 (93.0%)	2 (0.5%)	29 (6.6%)
Ceftriaxone	422	387 (91.7%)	0 (0.0%)	35 (8.3%)
Ceftolozane/Tazobactam	364	364 (100.0%)	0 (0.0%)	0 (0.0%)
Cefpodoxime	327	269 (82.3%)	0 (0.0%)	58 (17.7%)
Netilmicin	309	304 (98.4%)	0 (0.0%)	5 (1.6%)
Cefaclor	162	123 (75.9%)	0 (0.0%)	39 (24.1%)
Aztreonam	132	115 (87.1%)	0 (0.0%)	17 (12.9%)
Piperacillin	132	48 (36.4%)	1 (0.8%)	83 (62.9%)
Ticarcillin/Clavulanic Acid	132	98 (74.2%)	13 (9.8%)	21 (15.9%)
Cefoxitin	131	122 (93.1%)	4 (3.1%)	5 (3.8%)
Cefuroxime Axetil	131	89 (67.9%)	7 (5.3%)	35 (26.7%)
Levofloxacin	131	78 (59.5%)	5 (3.8%)	48 (36.6%)
Minocycline	131	96 (73.3%)	11 (8.4%)	24 (18.3%)

Antibiotic	n tested	Susceptible, n (%)	Susceptible, Increased Exposure, n (%)	Resistant, n (%)
Moxifloxacin	131	83 (63.4%)	0 (0.0%)	48 (36.6%)
Nalidixic Acid	131	76 (58.0%)	0 (0.0%)	55 (42.0%)
Ofloxacin	131	80 (61.1%)	1 (0.8%)	50 (38.2%)
Tetracycline	131	63 (48.1%)	0 (0.0%)	68 (51.9%)
Ticarcillin	128	46 (35.9%)	0 (0.0%)	82 (64.1%)
Tigecycline	128	128 (100.0%)	0 (0.0%)	0 (0.0%)
Cefalotin	127	63 (49.6%)	24 (18.9%)	40 (31.5%)
Chloramphenicol	122	86 (70.5%)	23 (18.9%)	13 (10.7%)
Colistin	108	1 (0.9%)	107 (99.1%)	0 (0.0%)
Trimethoprim	55	31 (56.4%)	0 (0.0%)	24 (43.6%)
Cefoperazone/Sulbactam	39	39 (100.0%)	0 (0.0%)	0 (0.0%)

Supplementary Table S4. Antimicrobial susceptibility profile for *Klebsiella pneumoniae* (isolate-level analysis)

Antibiotic	n tested	Susceptible, n (%)	Susceptible, Increased Exposure, n (%)	Resistant, n (%)
Amoxicillin/Clavulanic Acid	162	93 (57.4%)	18 (11.1%)	51 (31.5%)
Amikacin	160	135 (84.4%)	13 (8.1%)	12 (7.5%)
Ampicillin	160	1 (0.6%)	0 (0.0%)	159 (99.4%)
Cefepime	160	88 (55.0%)	0 (0.0%)	72 (45.0%)
Cefotaxime	160	78 (48.8%)	1 (0.6%)	81 (50.6%)
Ceftazidime	160	82 (51.2%)	1 (0.6%)	77 (48.1%)
Ciprofloxacin	160	75 (46.9%)	12 (7.5%)	73 (45.6%)
Gentamicin	160	102 (63.8%)	4 (2.5%)	54 (33.8%)
Imipenem	160	134 (83.8%)	6 (3.8%)	20 (12.5%)
Meropenem	160	136 (85.0%)	2 (1.2%)	22 (13.8%)
Trimethoprim/Sulfamethoxazole	160	92 (57.5%)	0 (0.0%)	68 (42.5%)
Piperacillin/Tazobactam	157	98 (62.4%)	15 (9.6%)	44 (28.0%)
Nitrofurantoin	156	41 (26.3%)	58 (37.2%)	57 (36.5%)
Ertapenem	143	132 (92.3%)	3 (2.1%)	8 (5.6%)
Cefixime	111	60 (54.1%)	0 (0.0%)	51 (45.9%)
Cefuroxime	91	27 (29.7%)	1 (1.1%)	63 (69.2%)
Fosfomycin	75	63 (84.0%)	1 (1.3%)	11 (14.7%)
Tobramycin	72	54 (75.0%)	0 (0.0%)	18 (25.0%)
Norfloxacin	66	3 (4.5%)	0 (0.0%)	63 (95.5%)

Antibiotic	n tested	Susceptible, n (%)	Susceptible, Increased Exposure, n (%)	Resistant, n (%)
Ceftriaxone	65	39 (60.0%)	0 (0.0%)	26 (40.0%)
Ceftazidime/Avibactam	49	49 (100.0%)	0 (0.0%)	0 (0.0%)
Netilmicin	46	42 (91.3%)	0 (0.0%)	4 (8.7%)
Cefpodoxime	40	26 (65.0%)	0 (0.0%)	14 (35.0%)
Ceftolozane/Tazobactam	34	34 (100.0%)	0 (0.0%)	0 (0.0%)
Cefaclor	31	6 (19.4%)	0 (0.0%)	25 (80.6%)
Aztreonam	26	14 (53.8%)	0 (0.0%)	12 (46.2%)
Cefoxitin	26	17 (65.4%)	0 (0.0%)	9 (34.6%)
Cefuroxime Axetil	26	12 (46.2%)	1 (3.8%)	13 (50.0%)
Levofloxacin	26	11 (42.3%)	1 (3.8%)	14 (53.8%)
Minocycline	26	12 (46.2%)	4 (15.4%)	10 (38.5%)
Moxifloxacin	26	12 (46.2%)	1 (3.8%)	13 (50.0%)
Nalidixic Acid	26	10 (38.5%)	0 (0.0%)	16 (61.5%)
Ofloxacin	26	11 (42.3%)	0 (0.0%)	15 (57.7%)
Piperacillin	26	0 (0.0%)	0 (0.0%)	26 (100.0%)
Tetracycline	26	14 (53.8%)	0 (0.0%)	12 (46.2%)
Ticarcillin/Clavulanic Acid	26	13 (50.0%)	2 (7.7%)	11 (42.3%)
Cefalotin	24	11 (45.8%)	1 (4.2%)	12 (50.0%)
Chloramphenicol	24	14 (58.3%)	1 (4.2%)	9 (37.5%)
Ticarcillin	24	0 (0.0%)	0 (0.0%)	24 (100.0%)
Tigecycline	22	17 (77.3%)	2 (9.1%)	3 (13.6%)
Colistin	19	0 (0.0%)	15 (78.9%)	4 (21.1%)
Trimethoprim	12	7 (58.3%)	0 (0.0%)	5 (41.7%)

Supplementary Table S5. Antimicrobial susceptibility profile for *Enterococcus faecalis* (isolate-level analysis)

Antibiotic	n tested	Susceptible, n (%)	Susceptible, Increased Exposure, n (%)	Resistant, n (%)
Ampicillin	109	109 (100.0%)	0 (0.0%)	0 (0.0%)
Linezolid	109	104 (95.4%)	3 (2.8%)	2 (1.8%)
Teicoplanin	109	109 (100.0%)	0 (0.0%)	0 (0.0%)
Gentamicin High Level (synergy)	108	47 (43.5%)	0 (0.0%)	61 (56.5%)
Streptomycin High Level (synergy)	108	52 (48.1%)	0 (0.0%)	56 (51.9%)
Tigecycline	108	108 (100.0%)	0 (0.0%)	0 (0.0%)

Antibiotic	n tested	Susceptible, n (%)	Susceptible, Increased Exposure, n (%)	Resistant, n (%)
Vancomycin	107	107 (100.0%)	0 (0.0%)	0 (0.0%)
Ciprofloxacin	103	44 (42.7%)	1 (1.0%)	58 (56.3%)
Erythromycin	103	0 (0.0%)	0 (0.0%)	103 (100.0%)
Tetracycline	103	7 (6.8%)	0 (0.0%)	96 (93.2%)
Azithromycin	17	0 (0.0%)	0 (0.0%)	17 (100.0%)
Imipenem	1	0 (0.0%)	1 (100.0%)	0 (0.0%)

Supplementary Table S6. Distribution of ESBL-producing Gram-negative isolates by pathogen

Pathogen	Total GN isolates, n	ESBL-positive, n (%)	ESBL-negative, n (%)
Escherichia coli	970	165 (17.0)	805 (83.0)
Klebsiella pneumoniae	157	57 (36.3)	100 (63.7)
Proteus mirabilis	41	0 (0.0)	41 (100.0)
Pseudomonas aeruginosa	22	0 (0.0)	22 (100.0)
Serratia marcescens	19	0 (0.0)	19 (100.0)
Enterobacter cloacae complex	17	0 (0.0)	17 (100.0)
Citrobacter freundii	13	0 (0.0)	13 (100.0)
Klebsiella oxytoca	10	1 (10.0)	9 (90.0)
Acinetobacter baumannii complex	4	0 (0.0)	4 (100.0)
Enterobacter aerogenes	4	0 (0.0)	4 (100.0)
Morganella morganii ssp morganii	4	0 (0.0)	4 (100.0)
Raoultella planticola	4	0 (0.0)	4 (100.0)
Citrobacter koseri	3	0 (0.0)	3 (100.0)
Enterobacter asburiae	3	0 (0.0)	3 (100.0)
Enterobacter cancerogenus	2	0 (0.0)	2 (100.0)
Myroides spp	2	0 (0.0)	2 (100.0)
Providencia stuartii	2	0 (0.0)	2 (100.0)
Alcaligenes faecalis ssp faecalis	1	0 (0.0)	1 (100.0)
Morganella morganii ssp sibonii	1	0 (0.0)	1 (100.0)
Pantoea agglomerans	1	0 (0.0)	1 (100.0)
Proteus hauseri	1	0 (0.0)	1 (100.0)
Raoultella ornithinolytica	1	0 (0.0)	1 (100.0)
Salmonella group	1	0 (0.0)	1 (100.0)
Salmonella ser. Typhi	1	0 (0.0)	1 (100.0)
Serratia fonticola	1	0 (0.0)	1 (100.0)

Pathogen	Total GN isolates, n	ESBL-positive, n (%)	ESBL-negative, n (%)
Stenotrophomonas maltophilia	1	0 (0.0)	1 (100.0)
Vibrio alginolyticus	1	0 (0.0)	1 (100.0)
